# Supplementary material for: The Fennoscandian Shield deep terrestrial virosphere suggests slow motion ‘boom and burst’ cycles
Source: Commun Biol. 2021 Mar 8;4:307. doi: 10.1038/s42003-021-01810-1 (PMC7940616; doi:10.1038/s42003-021-01810-1)
Supplement: Supplementary file 2 — Supplementary Information [file 42003_2021_1810_MOESM2_ESM.pdf]

The Fennoscandian Shield deep terrestrial virosphere suggests slow motion 'boom and burst' cycles

Karin Holmfeldt, Emelie Nilsson, Domenico Simone, Margarita Lopez-Fernandez,

Xiaofen Wu, Ino de Bruijn, Daniel Lundin, Anders F. Andersson,

Stefan Bertilsson and Mark Dopson

Supplementary Files

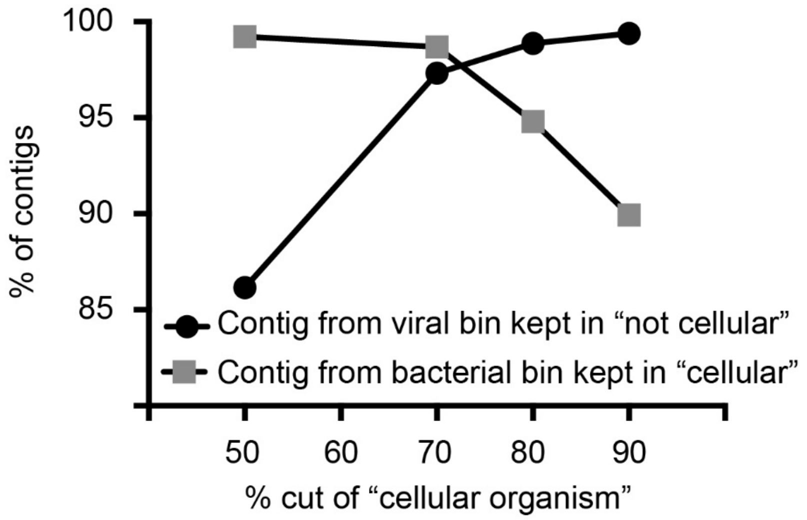

Supplementary Figure 1. Defining the cut-off used to verify likely viral contigs. Comparison of two different methods to define if a contig is of viral origin. Black circles show the percentage of contigs that occurred in both a viral bin and "non-cellular' while grey squares show the percentage of contigs that occurred both in a bacterial bin and in "cellular' fractions.

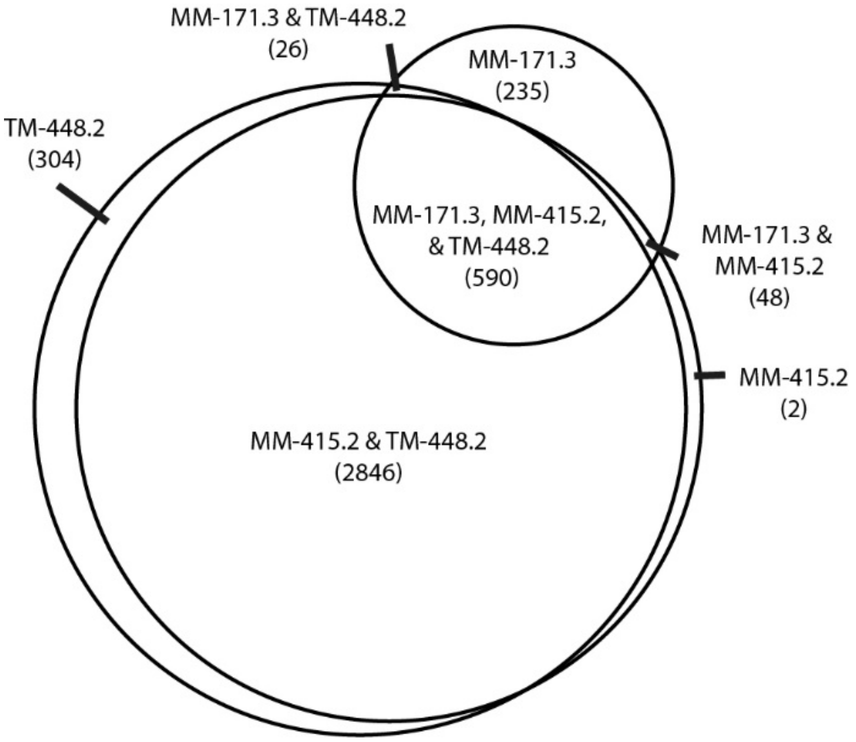

Supplementary Figure 2. Presence of viral contigs in the different groundwaters. Semi-quantitative Venn diagram of presence/absence of the coverage of the MM-171.3, MM-415.2, and TM-448.2 metagenome reads on the contigs.

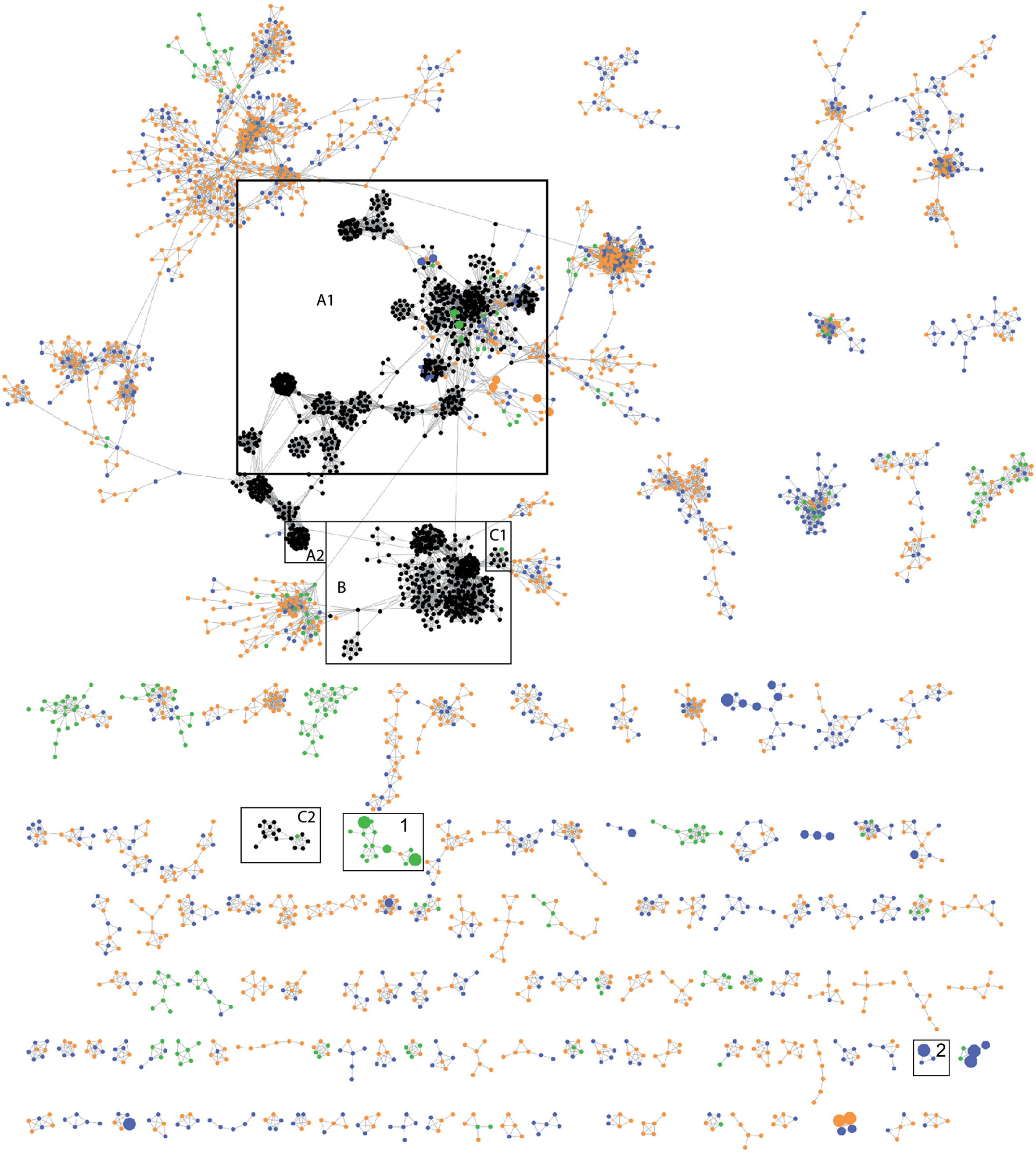

Supplementary Figure 3. Network showing the connection between Äspö HRL contigs originating from different groundwaters and to viral isolates. A selected portion of the network analysis of the contigs from the MM-171.3 (green), MM-415.2 (orange), and TM-448.2 (blue) groundwaters along with sequences from bacterial and archaeal viral isolates in the NCBI RefSeq database (black). Boxes marked with letters refer to phages that mainly infect Gamma-, Beta-, and Alphaproteobacteria (A1 & A2); Firmicutes (B); and Bacteriodetes

(C1 & C2). Boxes marked with numbers refer to clusters consisting of viral contigs both in the core and in individual groundwaters. Node sizes are as described in Fig. 1. Some unconnected networks have been moved due to spatial considerations and contig names in the Cytoscape file are defined as MM-171.3 = MM, MM-415.2 = UM, and TM-448.2 = OS. The complete and unedited Cytoscape figure is available in Figshare[1],

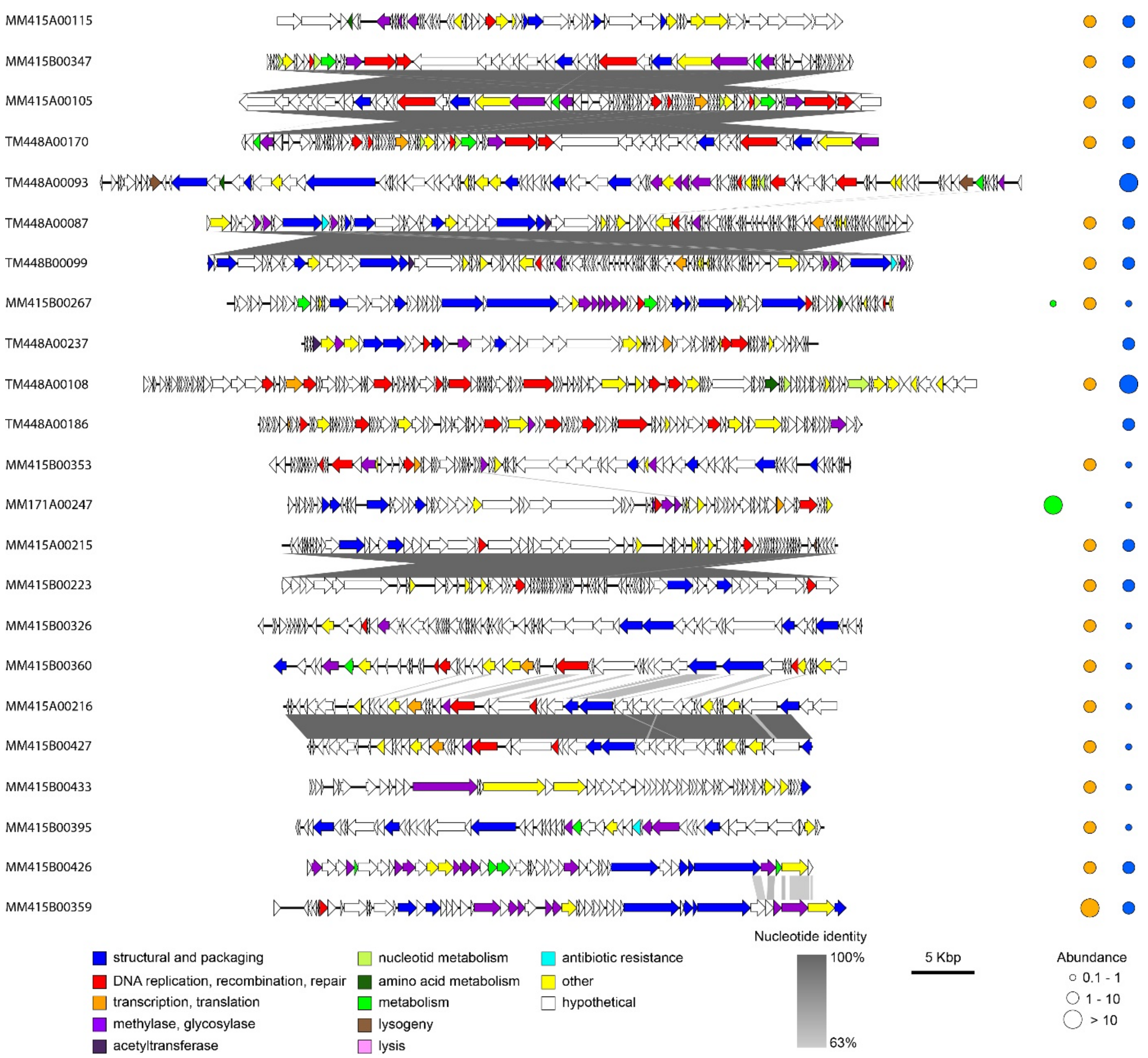

Supplementary Figure 4. Annotation of viral contigs potentially infecting Firmicutes and their relative abundance within the Äspö HRL groundwaters. All viral contigs >40 kb suggested by kmer analysis to infect Firmicutes in the three Äspö HRL groundwaters. Functional annotation of the predicted genes is given by color coding, nucleotide identity between contigs is given by the heat scale, the scale bar represents contig length, and abundance is given as mean base pair read depth normalized for the metagenome size in the MM-171.3 (green), MM-415.2 (orange), and TM-448.2 (blue) groundwaters.

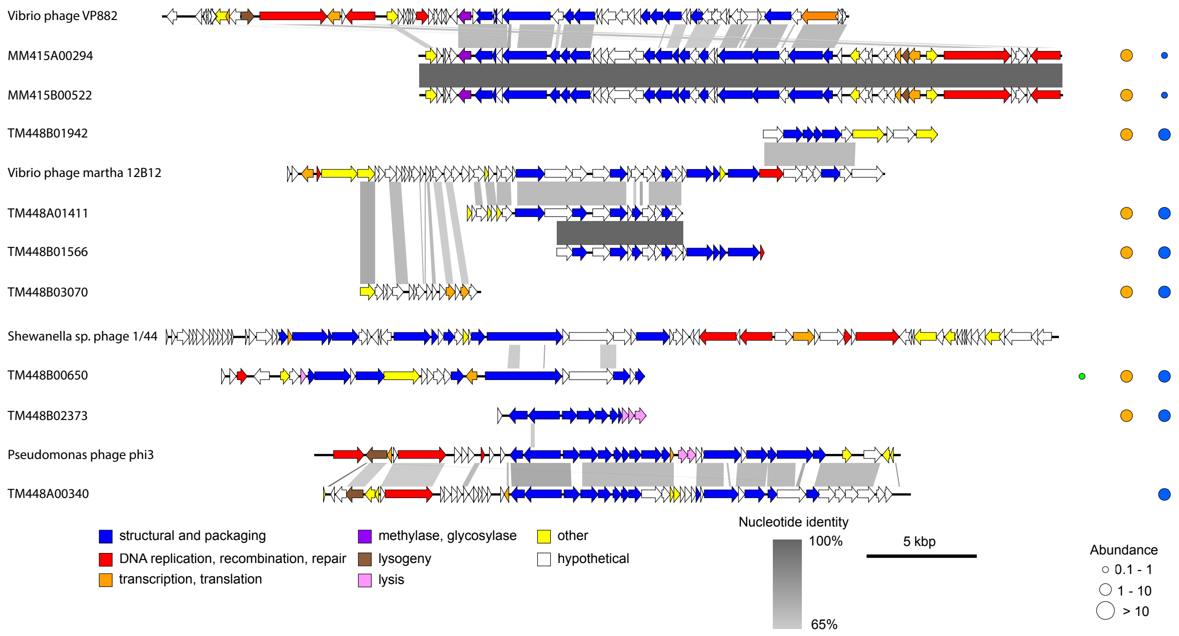

Supplementary Figure 5. Annotation of viral contigs similar to known Gammaproteobacterial phages and their relative abundance within the Äspö HRL groundwaters. Viral contigs sharing a relatively high proportions of protein similarity to previously isolated phages infecting Gammaproteobacterial hosts, visualized with shared nucleotide identity. Functional annotation of the predicted genes is given by color coding, nucleotide identity between contigs is given by the heat scale, the scale bar represents contig length, and abundance is given as mean base pair read depth normalized for the metagenome size in the MM-171.3 (green), MM-415.2 (orange), and TM-448.2 (blue) groundwaters.

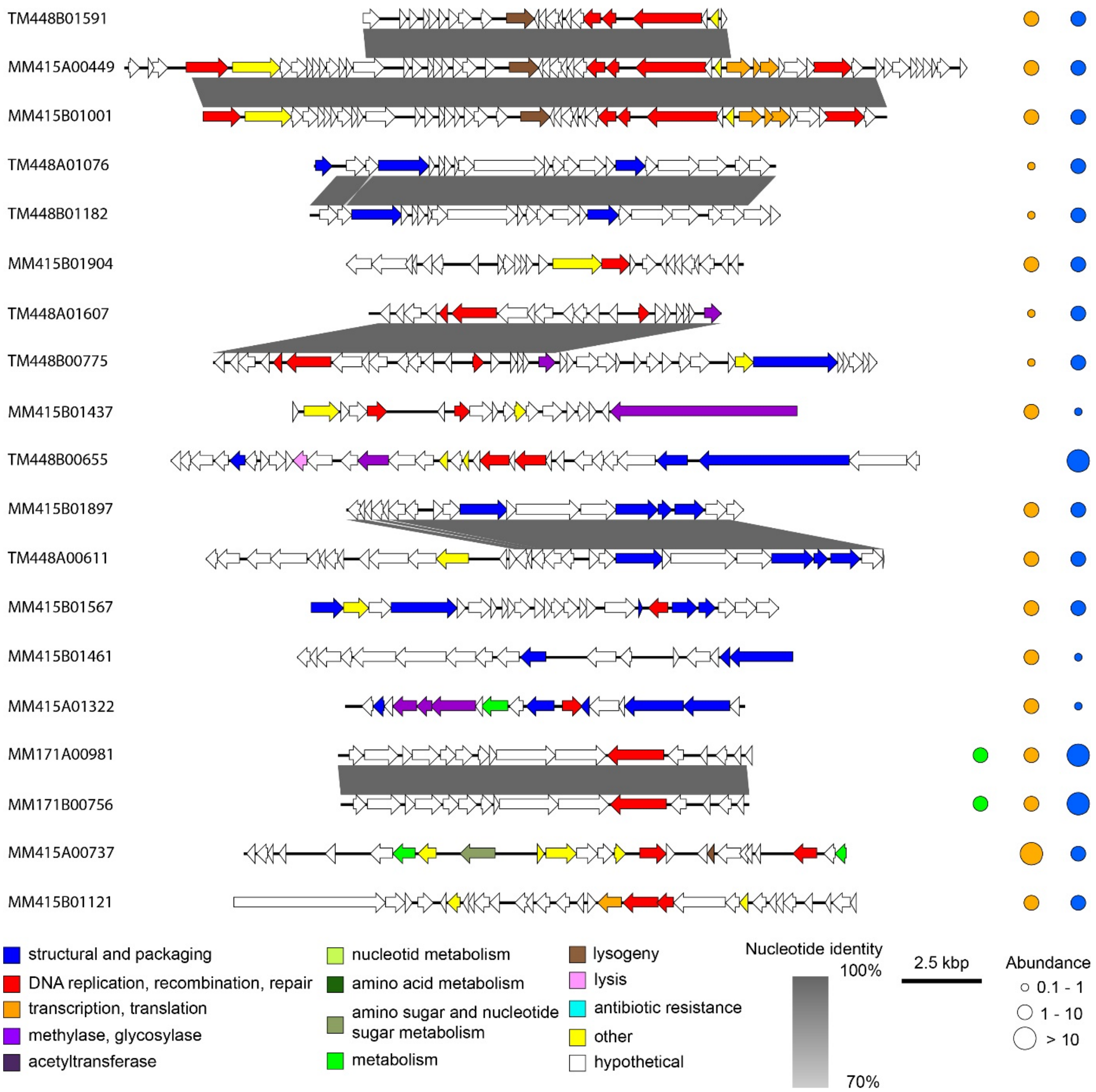

Supplementary Figure 6. Annotation of viral contigs potentially infecting Desulfobacteriota and their relative abundance within the Äspö HRL groundwaters. All viral contigs >10 kb suggested by kmer analysis to infect Desulfobacteriota in the three Äspö HRL groundwaters. Functional annotation of the predicted genes is given by color coding, nucleotide identity between contigs is given by the heat scale, the scale bar represents contig length, and abundance is given as mean base pair read depth normalized for the metagenome size in the MM-171.3 (green), MM-415.2 (orange), and TM-448.2 (blue) groundwaters.

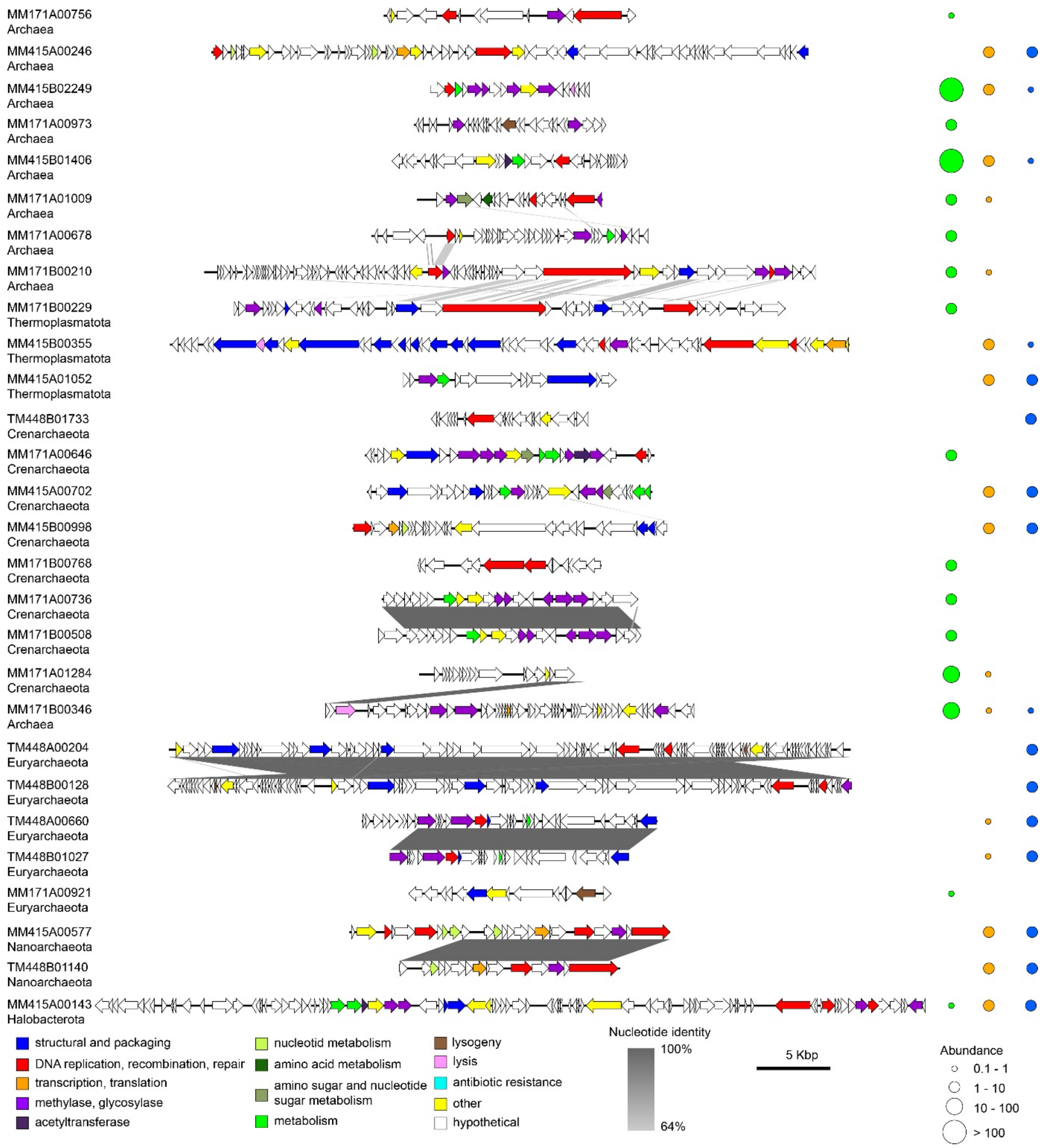

Supplementary Figure 7. Annotation of viral contigs potentially infecting Archaea and their relative abundance within the Äspö HRL groundwaters. All viral contigs >10 kb suggested by kmer analysis to infect Archaea in the three Äspö HRL groundwaters. Functional annotation of the predicted genes is given by color coding, nucleotide identity between contigs is given by the heat scale, the scale bar represents contig length, and abundance is given as mean base pair read depth normalized for the metagenome size in the MM-171.3 (green), MM-415.2 (orange), and TM-448.2 (blue) groundwaters.

Supplementary Table 1. Basic metagenome information for the Äspö HRL groundwaters. Sequencing information for the individual metagenomes including metagenome size, number of viral contigs, reads recruited to viral contigs as part of whole dataset, and % ORFs with matches to NCBI nr and ViralDB.

Metagenome	Metagenome ID in Wu et al.[2]	Metagenome size (Gbp)	Metagenome size (# Mreads pairs)	# viral contigs	# ORFs	Reads recruited to viral contigs as part of whole dataset (%)	Reads recruited to bacterial contigs as part of whole dataset (%)	% ORFs with matches to NCBI nr (June 2017)	% ORFs with matches to cellular organisms in NCBI nr (June 2017)	% ORFs with matches to viruses in NCBI nr (June 2017)	% ORFs with matches to ViralDB (July 2019)	Coverage estimates (%) from Nonpareil
MM-171.3_A	MMS_A	16.38	81.09	190	4001	1.7	45.50	45	44	1	25	85
MM-171.3_B	MMS_B	15.75	77.98	221	4220	1.9	41.32	41	40	1	24	83
MM-415.2_A	UMS_A	10.85	53.70	969	17707	4.69	9.10	40	37	3	24	66
MM-415.2_B	UMS_B	15.85	78.49	1453	26185	4.61	12.76	40	37	3	24	72
TM-448.4_A	OSS_A	13.75	68.06	618	11545	6.48	11.88	40	37	3	23	77
TM-448.4_B	OSS_B	15.75	75.00	600	11208	4.63	16.29	40	37	3	23	79

References

1. Lundin, D. & Holmfeldt, K. The deep terrestrial virosphere. Figshare, DOI: 10.6084/m6089.figshare.11590494.v11590491 (2020).
2. Wu, X. et al. Microbial metagenomes from three aquifers in the Fennoscandian shield terrestrial deep biosphere reveal metabolic partitioning among populations. ISME J 10, 1192-1203 (2015).
3. Kadnikov, V. V. et al. Genomes of three bacteriophages from the deep subsurface aquifer. Data in Brief 22, 488-491, doi:https://doi.org/10.1016/j.dib.2018.12.045 (2019).
4. Dupont, C. L. et al. Functional tradeoffs underpin salinity-driven divergence in microbial community composition. PloS One 9, e89549, doi:10.1371/journal.pone.0089549 (2014).
5. Hurwitz, B. L. & Sullivan, M. B. The Pacific Ocean Virome (POV): A marine viral metagenomic dataset and associated protein clusters for quantitative viral ecology. PloS One 8, e57355, doi:10.1371/journal.pone.0057355 (2013).
6. Chow, C. E., Winget, D. M., White, R. A., 3rd, Hallam, S. J. & Suttle, C. A. Combining genomic sequencing methods to explore viral diversity and reveal potential virus-host interactions. Front. Microbiol. 6, 10.3389/fmicb.2015.00265 (2015).
7. Tangherlini, M., Dell'Anno, A., Zeigler Allen, L., Riccioni, G. & Corinaldesi, C. Assessing viral taxonomic composition in benthic marine ecosystems: reliability and efficiency of different bioinformatic tools for viral metagenomic analyses. Sci. Rep. 6, 10.1038/srep28428 (2016).

8. Sible, E. et al. Survey of viral populations within Lake Michigan nearshore waters at four Chicago area beaches. Data in Brief 5, 9-12, doi:https://doi.org/10.1016/j.dib.2015.08.001 (2015).
9. Nilsson, E. et al. Genomic and seasonal variations among aquatic phages infecting the Baltic Sea Gammaproteobacterium Rheinheimera sp. strain BAL341. Appl. Environ. Microbiol. 85, doi:10.1128/aem.01003-19 (2019).
10. Hugerth, L. W. et al. Metagenome-assembled genomes uncover a global brackish microbiome. Genome Biol. 16, 279, doi:10.1186/s13059-015-0834-7 (2015).
11. Brum, J. R. et al. Illuminating structural proteins in viral "dark matter" with metaproteomics. Proc Natl Acad Sci USA 113, 2436-2441 (2016)
