# Supplementary material for: The Fennoscandian Shield deep terrestrial virosphere suggests slow motion ‘boom and burst’ cycles
Source: Commun Biol. 2021 Mar 8;4:307. doi: 10.1038/s42003-021-01810-1 (PMC7940616; doi:10.1038/s42003-021-01810-1)
Supplement: Supplementary file 3 — Description of Additional Supplementary Files [file 42003_2021_1810_MOESM3_ESM.pdf]

Description of Additional Supplementary Files

File Name: Supplementary Data 1

Description: Details of each contig in the respective metagenomes defined to be of viral origin. Detailed information for each contig including accession number, length, number of genes, similarity to different databases, predicted host, and abundance and activity in the various Äspö HRL groundwaters.

File Name: Supplementary Data 2

Description: Details of comparison to publicly available metagenomic datasets used in Figure 2. Sheet1 contains the best match of each protein in the Äspö HRL dataset to the individual publicly available datasets while Sheet2 contains details of the metagenomic datasets, including source, environment, and accession number.

File Name: Supplementary Data 3

Description: Relative abundance of the Äspö HRL viral contigs in Baltic Sea metagenomes. Mapping of Linnaeus Marine Observatory (LMO) metagenome reads to the Äspö HRL contigs. Sheet “recruit. LMO viral metag.” reports the mapping results of LMO viral metagenomes to individual Äspö HRL viral contigs. Sheet “recruit. LMO micro. metag.” is a summary of the recruitment of LMO microbial metagenomes to all Äspö HRL contigs, and sheet “LMO accession number” provides the accession numbers of the LMO metagenomes used.

File Name: Supplementary Data 4

Description: Functional and taxonomic annotation for each protein in the Äspö HRL viral dataset. Details of best match of Äspö HRL viral ORFs to PFAM (April 2018), NCBI nr (June 2017), Viral DB (July 2019), and marine viral proteome dataset.
